# Primary School Teachers’ Conceptions of Reading Comprehension Processes and Its Formulation

**DOI:** 10.3389/fpsyg.2020.00615

**Published:** 2020-05-07

**Authors:** Xinhua Zhu, Choo Mui Cheong, Guan Ying Li, Jacqueline Wu

**Affiliations:** ^1^Department of Chinese and Bilingual Studies, The Hong Kong Polytechnic University, Kowloon, Hong Kong; ^2^Faculty of Education, University of Hong Kong, Pokfulam, Hong Kong; ^3^Center for Teaching and Learning Development, National Taiwan University, Taipei, Taiwan

**Keywords:** reading comprehension processes, teachers’ conception, sense-making, influencing factors, Chinese language

## Abstract

Understanding reading comprehension processes is vital for teachers to effectively conduct teaching and assessment of reading in schools. Hong Kong students’ outstanding reading performance internationally is commonly attributed to the implementation of reading comprehension models in its Chinese language curriculum, however, the understanding of teachers’ conceptions of reading comprehension is still very limited. This phenomenographic study interviewed 28 in-service Chinese language teachers from eight Hong Kong primary schools and illustrated the elicited conceptions of a model of reading comprehension processes (i.e., Six Types of Reading Comprehension Process Model, a widely used model in Hong Kong). The three categories of teachers’ conceptions found are as follows: understanding basic ideas of a text, sequential development of holistic comprehension skills, and fostering independent readers. This study further employed sense-making theory to investigate the formulation of the teachers’ conceptions. It is found that the conceptions were shaped when teachers’ conventional practices that focused on rote-learning interplayed with the continual regulating means of the implementation. Teachers are also highly influenced by factors like attainability by their students and agreement with own philosophy. This study also implied that teachers’ beliefs will need to be brought to light in order for new learning to happen.

## Introductory Background

Reading competence is anchored as a foundational element in primary and secondary education. Proficient readers construct meaning from text by using higher-order thinking skills as opposed to less proficient readers who rely solely on retrieving explicit or factual information from the text in their comprehension processes (Pearson and Cervetti, 2015). In order to cultivate higher-order reading skills, researchers introduce reading comprehension models to guide instruction and assessment. Although these models have been devised to capture reading processes, teachers often only have limited knowledge about them.

The rapidly growing research on teachers’ conception contributed richly to how teachers’ personal knowledge of the subject matter influenced their everyday practice (Kagan, 1992; Calderhead and Berliner, 1996). Despite the efforts made to investigate practices in reading classrooms, there have been few qualitative accounts of how the teachers’ conceptions of reading are formulated. Applying phenomenographic research, we intend to unveil a hierarchy of categories of teachers’ conception, and examining teachers’ sense-making processes will provide insights on curriculum initiatives in schools (Coburn, 2001; Maitlis and Christianson, 2014; Pyhältö et al., 2018). Incorporating the above two research strands offers a new avenue to unveil teachers’ conceptions on a reading comprehension process model most widely used in Hong Kong. The insights on teachers’ beliefs and the formation of these varied conceptions will bring to light the potential problems and provide suggestions to resolve their concerns.

### Use of Reading Comprehension Models in Literacy Classrooms

Reading is a dynamic and complex process that includes thinking, reasoning, imagining, and interpreting (Van den Broek et al., 1999). According to Kintsch (1988, 1998), the process of comprehension can be understood as the construction of a mental representation of the text in a situation model through the integration of meaning from the text. Similarly, Pecjak et al. (2011) discussed the cognitive factors of reading comprehension as “the interactional process between text, background knowledge (content knowledge and text characteristics knowledge), reading context, motives, and goals” (p. 54).

Researchers also took a perspective of the classification of skills to capture the complex processes of reading comprehension, which optimized the opportunity for effective implementation in literacy classrooms. Both Zhu’s (2005a,b) and Afflerbach et al. (2015, 2011) work referred to the revised Bloom’s Taxonomy of cognitive outcomes of students’ learning (Anderson et al., 2001; Krathwohl, 2002) and suggested that whereas basic thinking skills are sufficient to decode text, higher-order thinking skills are required to deduce and infer meaning to form idea units from the text. Afflerbach and colleagues (2011, 2015) depicted higher-order thinking skills as goal directed, responsive, and self-regulated. Zhu (2005a) detailed the six cognitive levels, that is, Six Types of Reading Comprehension Process Model, shortened as the Six Types, around local and global reading. This hierarchy of cognitive processes is defined as follows: (1) *Retrieving*: extracting information; (2) *Explaining*: clarifying with information from the text; (3) *Summarizing*: identifying and organizing the main ideas of the text; (4) *Elaborating*: inferring implicit meanings and ideas; (5) *Evaluating*: appraising and critiquing content and textual elements; and (6) *Creating*: suggesting new ways of thinking. *Retrieving* and *Explaining* specify the local content of the text explicitly, whereas *Summarizing*, *Elaborating*, *Evaluating*, and *Creating* deal with wider scope across the text read. We noted that both frameworks emphasized the cultivation of higher-order thinking (i.e., the last four in the Six Types) in reading. These efforts are in line with international and national curriculum and assessment programs that meet new literacy needs (Mullis et al., 2016). Moreover, researchers attributed the excellent performance by Hong Kong students in Programme for International Student Assessment (PISA) as partly a result of the implementation of the Six Types (e.g., Lau, 2009).

Although there is a long-standing body of reading comprehension research and effortful connection to instructional practices, it is alarming to find that teachers are only minimally incorporating the reading frameworks into their curricula (Alfassi, 2004; Harvey and Goudvis, 2013). They completely omitted explicit teaching or facilitated their students to apply the various reading comprehension strategies (Pressley et al., 1998) and engage their students in activities like reading aloud rather than conducting high cognitive activities (Nazari and Bagheri, 2014). By holding back the teaching, students are cut off from the opportunity to attain higher-order skills. As many studies have been conducted on students’ performance (e.g., Pressley et al., 1998; MacMillan, 2014), there is a need for more research into the subject from the perspectives of teachers’ understanding.

### Teachers’ Conception of Reading and Phenomenographic Study

It is generally assumed that teachers’ conceptions about teaching and learning, their students, and the subjects they teach inform their instructional practice (Kagan, 1992; Calderhead and Berliner, 1996). However, in an attempt to compare between US teachers and Argentina teachers, Taboada and Buehl (2012) found that although both groups of teachers are in partial agreement that reading comprehension should address higher levels of text understanding, they differ in their approach. The US teachers explicitly teach the reading strategies, but the Argentina teachers emphasized the importance of stimulation and encouragement of reading, without providing instructional support. Furthermore, although reading comprehension is carried out almost on a daily basis in language classrooms, research on teachers’ beliefs about reading comprehension is surprisingly limited. Applying the theory of teachers’ practical knowledge, Meijer et al. (1999) study found that teachers who focused on subject matter knowledge, student knowledge, or knowledge of student learning and understanding exhibited three typologies of practical knowledge, of which the third is most balanced. Note that the distinctive descriptions are not elicited typically from the teachers but are ideal types of practical knowledge articulated by the researchers. Grishma’s (2000) study observed a group of 12 teachers teaching reading over a 3-year period and found that teachers’ funds of knowledge (professional, practical, and personal) interacted in complex ways to affect their classroom practice. In Hong Kong, Lau studied teachers’ orientation of Chinese reading instruction and found a higher level of acceptance of the competence-based orientation after almost a decade since the implementation of the Six Types (Lau, 2007a, b). Interestingly, the teachers who reported strong beliefs in both competence- and text-based orientation employed competence-based instructional strategies more than did their counterparts who only believed in either one or the other type of orientation. These previous studies tended to base on some presuppositions, which may have neglected what the teachers really experienced or how they have perceived reading instruction. Moreover, they have yet to critically identify distinctive aspects or typologies. As teachers’ learning is viewed as changing their ways of seeing (Marton and Booth, 1997), it is essential to identify the different ways teachers perceive reading instruction in order to support their professional development. Rather than presuming that a particular way of professional support is effective, this study aimed to explore more openly how teachers actually went about teaching reading in primary schools, and thus, the approach of phenomenography was adopted.

Marton (1986) held the principle that “each phenomenon, concept, or principle can be understood in a limited number of qualitatively different ways” (p. 31). Based on this principle, phenomenography reveals the distinctive ways in which individuals experienced and discerned a phenomenon in its critical aspects. The varied discernments are further organized into categories of description, logically and hierarchically interrelated to establish a typology (Ashworth and Lucas, 1998). For instance, Prosser et al. (1994) described the qualitatively different ways that teachers saw what teaching meant to them in their subject areas as a hierarchical structure, whereas Tan (2013) identified teachers’ conception of alternative assessment as being conservative, pragmatic, and progressive beyond and across subjects. Focusing on the “experience-as-described” (Ashworth and Lucas, 1998, p. 415), phenomenographic investigation offers new insights into the varied teachers’ conceptions of reading that are typically distilled from the teachers’ teaching experiences.

### Factors of Teachers’ Conception and Sense-Making Theory

The literature on conceptual change in many different knowledge domains consistently suggests that personal beliefs function as both the filter and foundation of new knowledge. Kagan (1992) made an assumption that “a teacher’s belief of his or her profession is situated in three important ways: in context (it is related to specific groups of students), in content (it is related to particular academic material to be taught) and in person (it is embedded within the teacher’s unique belief system)” (p. 74). The introduction of educational reforms begins as a trigger that may deviate away from teachers’ original belief systems and conventional practices (Maitlis and Christianson, 2014). The sense-making theory (Coburn, 2001, 2005; Maitlis and Christianson, 2014; Pyhältö et al., 2018) investigates teachers’ meaning-making processes by which teachers construct and reconstruct multiple messages about their practice. Research implies that curriculum work that engages educational practitioners in the sense-making process are more likely to produce sustainable effects. One strand of research focuses on understanding the dynamic interaction of new messages challenging conventional thoughts to activate the sense-making process while the teachers are busy dealing with their daily routines. With regard to reading, this sense-making process inevitably makes teachers the active agents shaping a new understanding of function and teaching of reading, and not only the receivers of the new messages about reading instruction. This active formulation of their understanding of any reform (Maitlis and Christianson, 2014) potentially shapes their classroom practice over time (Pyhältö et al., 2018). Therefore, researchers see value in investigating how teachers react to messages about reading instruction.

The following understandings about teacher sense-making in reading instruction will help us in contextualizing the current study. Firstly, the reform policy acts as a trigger to the sense-making process and carries new messages of reading through various forms, including curriculum guides and other supporting documents, assessment systems, teacher professional development, textbooks and other materials, and professional development (Coburn, 2001; Pyhältö et al., 2018). These regulative means in the sense-making process (Maitlis and Christianson, 2014) take multiple forms and sources, and the teachers may be forming varied interpretations of them. Secondly, in order to understand the teachers’ sense-making process, we need to first acquire knowledge about teachers’ established thinking, which is formed in larger social, historical, and cultural contexts. Thus, it is important to take into account the past episodes and local context in combination with the trigger, that is, new messages about reading instruction (Maitlis and Christianson, 2014). Thirdly, Coburn’s (2001) study identified gatekeeping as the key process that teachers went through. There are four subcategories of gatekeeping: “attainability level by students” – does not apply to their grade level, too difficult for their students; “agreement with own philosophy” – philosophically opposed, does not fit; “feasibility due to resources issue” – unmanageable; and “comprehensibility” – completely outside the bounds of comprehensibility, did not feel they understand it.

## The Study

At the beginning of the 21st century, Hong Kong underwent a system-wide curricular reform (CDC, 2001; CDC et al., 2015), and the ability to read critically and innovatively took center stage. Although reading curriculum and public examinations used reading models like the Six Types to teach and assess students’ cognitive development (HKEAA, 2005; Zhu, 2005b; CDC, 2007), researchers still observed unsatisfactory development of students’ cognitive abilities to manage complex tasks such as critical thinking and evaluating in Chinese language classrooms (Lau and Chan, 2007). We still lack knowledge on whether teachers view reading comprehension as a literal understanding of a text or a process that involves readers, the text, and the social context. In particular, the backdrop of medium-term review of new literacy necessitates an investigation of the teachers’ conception on the purpose of their reading instruction and the extent of the attainment of higher-order thinking by their students (CDC and HKEAA, 2013), which may differ from the intent of the reading instruction (Zhu et al., 2016). The value gained from understanding teachers’ conceptions and their formulation can contribute to the investigation of the progress of the curriculum reform on the practical front. The research questions for this study are as follows:

1. What are the in-service Chinese language teachers’ conceptions of reading comprehension processes, for example, the Six Types?
2. What are the factors influencing these conceptions?

### Participants

Twenty-eight in-service Chinese language teachers from eight Hong Kong primary schools were recruited based on opportunity sampling. The distribution of the schools is as follows: five schools from the New Territories, two from Kowloon, and one from Hong Kong Island; this reflects the proportion of primary schools distributed geographically across Hong Kong. Teachers interviewed had majored in the Chinese language during their undergraduate or professional studies and are currently teaching Grade 3 and/or 4 at the time of the study. The observation that Grade 4 has been recognized as the cutting point between learning to read and reading to learn for students (Mullis et al., 2016) is a deciding factor for the selection criteria, along with the scope to recruit a wide spread of experience in the participants. There are 23 female and 5 male participants, with 9 of them having ≤ 5 years of teaching experience, 4 with 6–10 years of teaching experience, and the rest with > 10 years of teaching experience. Besides the class of Grade 3 or 4 students that the teachers are teaching, they either have another Chinese class or are teaching another subject. Therefore, they are in frequent contact with approximately 60 students each academic year. All participants have either attended workshops and seminars or participated in school-based sharing sessions on the Six Types before their participation in the study.

Selection of participants for phenomenographic study is not intended to be representative but to collectively elicit a range of variation of experiences of the phenomenon that can depict the range of meanings of the phenomenon for an intended audience (Åkerlind, 2002). As far as the participants are familiar with Six Types, they are considered to be able to address the general teaching fraternity in Hong Kong.

### Interview Design and Outline

The interview protocol used in this study was purposefully designed to guide the participants to reiterate their experience of the phenomenon. Open-ended probing questions were modified accordingly as the data collection progress to fit with the flow of the discourse throughout the interview and to elicit more meaningful exchanges and dialogues (Marton and Booth, 1997). Six questions and their follow-up queries were asked, eliciting the participants’ experiences on how they are using the Six Types in their practice and cultivating students’ higher-order thinking. For instance, the first question involves a card organization task, which will systematically elicit and represent conceptual structures of human beings’ understanding of their environment in terms of their own construct system (Calderhead and Berliner, 1996). Teachers are presented with the six key concepts concerning comprehension and asked to organize them into networks of relationships to reflect their own thinking.

A pilot study was conducted with a Grade 4 Chinese language teacher. The interview protocol was then adjusted according to the response and feedback provided by the participant. The final version of the interview protocol can be found in Supplementary Appendix A.

### Data Collection and Analysis

The interview data were recorded and transcribed verbatim for analysis alongside the audio files in order to capture the nuances expressed in colloquial Cantonese. The analysis was done in three stages by three researchers: (1) familiarizing with the data, (2) sorting into categories of description, and (3) establishing the hierarchical structure (Åkerlind, 2012). Each researcher completed stages 1 and 2 independently before meeting to discuss their findings to complete stage 3 together. Intersubjective agreement of the categories was ensured through discussions to settle any differences in opinion.

Our data analysis adopted Marton and Pang’s (2007) principle by denoting the global meaning of the object conceptualized, and we followed Marton and Booth’s (1997) method, which focuses on “identifying a small number of qualitatively distinct descriptive categories of the ways in which the subjects experience the phenomena of interest” (p. 138). We utilized the structural focus and transcript focus as an ongoing process in the analysis of the transcripts (Tan, 2013). For instance, one of the quotes mentioned that a teacher’s assessment blueprint included 70% questions on Retrieving, Explaining, and Summarizing; the quote was read against the surrounding context in the original transcript to confirm our interpretation. We found that the teacher also mentioned that she was not teaching Creating at all, which affirmed the point. At the same time, the quote might be related to the existing categories of description, for example, the focus on basic ideas of a text especially in an examination setting, and the quote would then be understood against the structure of the outcome space. The two foci are used simultaneously, intending to approximate the truthfulness of the results.

The validity of this method is also ensured with bracketing to dispose of any presuppositions that the researcher might have during the interviews regarding what the participants shared (Marton, 1986; Åkerlind, 2012; Forster, 2016). The researcher should not insert any subjective opinion during the interview to keep the trustworthiness and authenticity of the data from the second-order perspective. A high degree of intersubjective agreement between the categories and the use of an analytical framework can address the issue of reliability in phenomenographic studies (Marton, 1986; Cope, 2002). Ashworth and Lucas (2000) have concluded that empathetic feelings rather than technical rationality can bring subjectivity to scientific standards, and that can also ensure the reliability of phenomenography as a proper research method in mainstream social sciences.

## Results and Discussion

### Teachers’ Conception of Reading Comprehension

Three multilayered conceptions of scope and purposes of reading comprehension emerged from the data. The ascending categories expanded from a basic understanding of text-based information, through holistic development of reading comprehension skills, to the application of the reading to other subjects and contexts. As the categories advanced, teachers also expressed that their students (1) could only understand the basic ideas of a text, (2) need to develop the basic skills first and then subsequently attain the higher-order skills, and (3) foster skills to become independent readers. Table 1 shows the hierarchical structure of the three levels of purpose, focus, and skills taught in reading comprehension, whereas Table 2 provides a quotation to illustrate the distinctive categories.

**TABLE 1:** Outcome space of teachers’ conception of reading comprehension.

	Category 1	Category 2	Category 3
The purpose of reading comprehension	Understanding the basic ideas of a text	Developing holistic comprehension skills	Fostering independent readers
The focus of reading comprehension	Basic text-based information	Basic text-based information and higher-order thinking	Higher-order thinking
Skills involved	Retrieving, explaining, and summarizing	Retrieving, explaining, summarizing, elaborating, evaluating, and creating	Retrieving, explaining, summarizing, elaborating, evaluating, and creating

**TABLE 2:** Typical quotation for each category elicited from the data.

Category	Quotation
Category 1	*We would use a table to plan our assessment. 70% of the questions will test on Retrieving, Explaining and Summarizing, while the rest will take up 30%. (Teacher 101)*
Category 2	*(When asked what approaches are used to teach Elaborating) I would break down my question into simpler questions, and I would scaffold the students into answering the individual questions first, and then combine the answers to formulate the answer of the original Elaborating level of understanding. (Teacher 402)*
Category 3	*To me, Elaborating, Evaluating and Creating are interrelated. I would ask the students to write about their life as a after reading task*… *for example, writing poems to express their thoughts. The students love this task, as they get to express freely. (Teacher 401)*

#### Category 1: Reading Comprehension for Understanding the Basic Ideas of a Text

**FIGURE 1:**
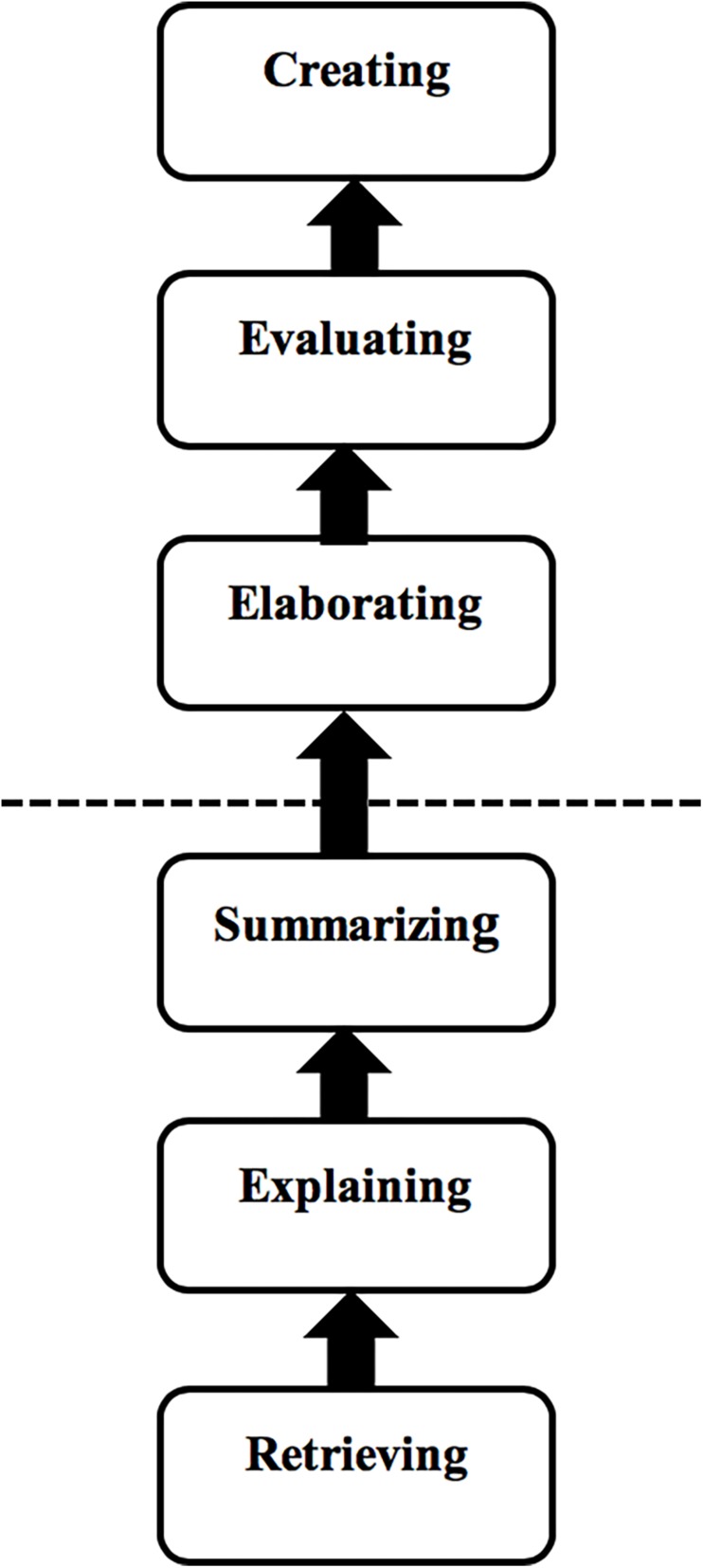
Linear process.

In the card organization task, Category 1 teachers structured the Six Types in a linear manner and separated them into two groups (see Figure 1). The first group consisted of Retrieving, Explaining, and Summarizing skills, and it was deemed essential to the basic understanding of a text. Many teachers indicated that lower graders generally perform well in identifying text-based information. The second group, comprised of Elaborating, Evaluating, and Creating skills, was considered to be more challenging and unachievable by lower graders. Teachers think that these higher-order skills are only achievable by students with high language proficiency or when they move up to higher grades, as the quotation below demonstrates:

I should be capable of, say guiding upper primary students to think through (higher-order questions), but I won’t be able to make it, really, with lower primary students. Basically, I focus on the first three skills and seldom tap into elaborating. (Teacher 804)

When asked to describe her instructional practices, another teacher, Teacher 102, also pointed out that Elaborating is a demanding challenge to her students because it requires more inferences to their own life experience. She felt that her students lacked adequate experience and the language to demonstrate a deeper understanding of the text. Teacher 801 further indicated that although she personally enjoyed designing creative tasks, she refrained from including them in a formal assessment. This was justified owing to the inability to guide students’ thoughts in line with assessment requirements. Thus, in order to reduce the risk of students failing, she explicitly expressed that she would focus on the basic skills instead in examinations.

I like creative tasks if I am preparing for my own teaching materials. However, I will back off if I have to assign grades…[It] is a risky choice, and I think it is easier to ask students to retrieve and summarize information in order to grade or guide them through the tasks. (Teacher 801)

Teachers felt that they are held accountable for students’ performance in assessment, and thus, they are having second thoughts about designing higher-order thinking tasks. The way teachers thought about creativity also reflects a lack of self-efficacy in evaluating creative thinking, resulting in that they tended to isolate creativity from other skills.

*I think creativity is detached from the text in terms of assessment and relevance. [*…*] We used to design creative tasks in the school, but it is difficult to grade students’ performance*… *[So] we try to avoid creative tasks in assessment now. (Teacher 903)*

The teachers’ understanding of the scope of learning to read is constrained largely by the foci of examinations. In order to avoid failing students and to ensure fairness and accountability, they placed greater emphasis on the basic cognitive skills rather than on higher-order thinking skills, especially during the lower grade stages.

#### Category 2: Reading Comprehension for the Sequential Development of Holistic Comprehension Skills

**FIGURE 2:**
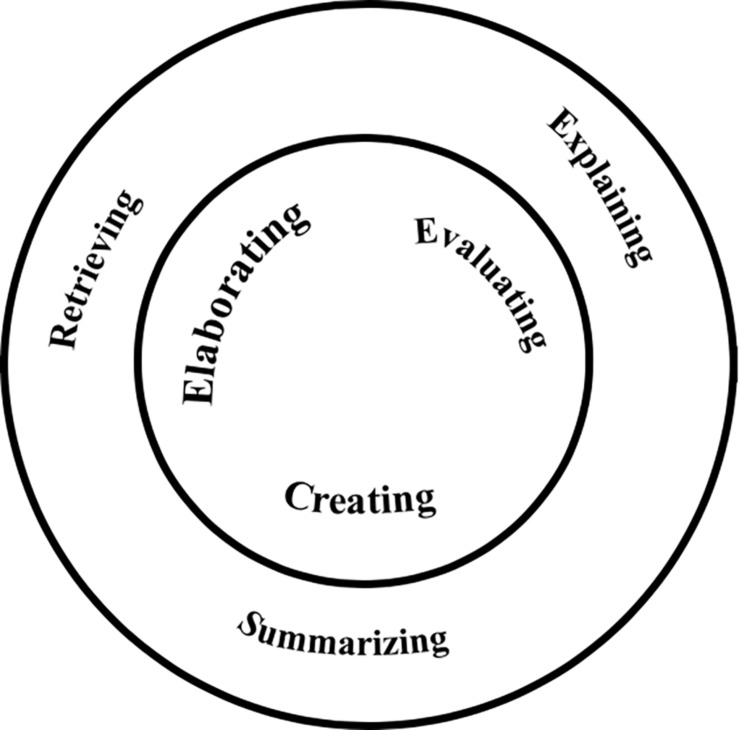
Dual-layer circle.

Category 2 teachers structured the Six Types similarly to Category 1 by classifying the Six Types into two groups but used a dual-layer circle instead (see Figure 2). The outer layer is the fundamental skills of reading comprehension and advances to the students’ higher-order thinking in the inner circle. The six skills are regarded as interdependent in the developmental process. The outer layer is used to support the acquisition of the core skills, as Teacher 603 pointed out that the ultimate goal of teaching reading comprehension is to enable students to demonstrate higher-order thinking skills building upon the basic understanding of the text.

You should not simply understand the superficial meaning of a text. Simply decoding the text and understanding its basic meaning will not allow you to get the gist of the author’s intent, to interact with the text, or to learn from the characters of the text. The latter part of the Six Types can help students to understand a text in a deeper manner and learn more. (Teacher 603)

Category 2 teachers tend to see the six skills as an indispensable, holistic developmental process; and they are all achievable by lower graders, which is supported by previous studies (e.g., Alfassi, 2004; MacMillan, 2014). Intervention experiments also proved that literacy instruction will successfully engage students in constructing and inferencing complex meanings from texts if it is guided by reading theories (Alfassi, 2004). It is also indicated that using questions to probe is essential to the development of students’ higher-order thinking skills. Even though the attempt might not always be successful, these questions can help teachers perform an ongoing evaluation of students’ understanding.

The two-layered model could be interpreted as the teachers seeing a sequential development between the layers. The outer layer serves as a foundation that is normally addressed in the beginning part of a reading lesson, and the understanding is accumulated and used to scaffold students to reach the ultimate goal of higher-order thinking skills.

#### Category 3: Reading Comprehension for Fostering Independent Readers

**FIGURE 3:**
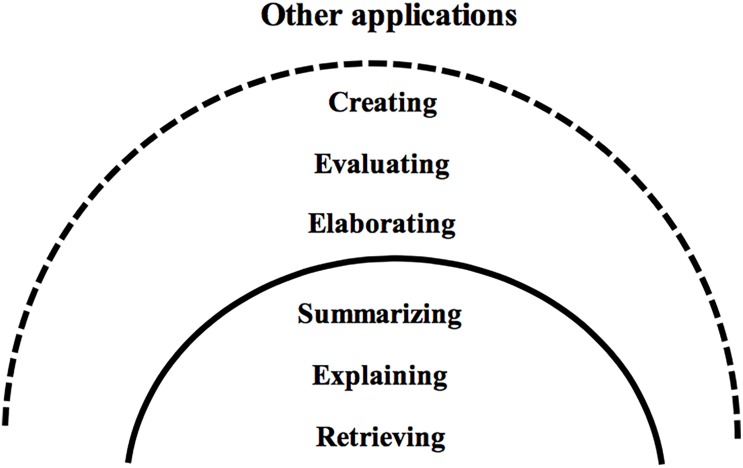
Applications of skills beyond reading.

In Category 3, the scope of reading comprehension encompasses learning to read and expands beyond, contrasting the focus on learning to read in Categories 1 and 2. As represented in Figure 3, reading instruction laid a firm foundation in reading classrooms for the application of knowledge and skills to different Key Learning Areas (CDC et al., 2015, p. 9). Teachers who attempted to expand learning beyond reading earned higher motivation and greater capacity from students to fulfill the desired learning outcomes.

Notably, teachers see the interconnectedness between academic disciplines found in higher-order thinking skills, and the application of higher-order thinking skills comes to the forefront of teachers’ conceptions. They focused on incorporating reading comprehension skills into subjects such as General Studies to enable students to complete assignments such as writing a newspaper commentary. Teacher 604 explained that a mastery of the Chinese language allows students to access wisdom from the life experiences of others and will, in turn, broaden students’ perspectives. She shared on a reading program that has been implemented in her school, which encouraged the sharing of personal thoughts and learning among peers. Based on her observations, students enjoyed reading and writing more in this program than in a language classroom.

*All [the] students*…*can read each other’s work [for the “On a Colorful Journey (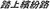)” project] and this is really helpful to them*… *It is different from what they usually do in a regular composition activity where there are too many constraints. They are allowed to write freely and I think their performance is better than usual.*

Reading comprehension is integrated with composition writing and learning in other subjects in this category. The goal of reading comprehension is to cultivate students’ reading competencies and benefit their future learning. The teachers’ expansion of the function of reading is also proved quantitatively in García-Madruga et al.’s (2014) study, which reported that students’ reading comprehension competence contributes significantly to their mathematics achievement. Teachers in this category illustrated their tendency to see reading comprehension as a cross-disciplinary approach to learning rather than exclusive to language learning. This reflects a shift in the focus of reading comprehension from learning to read to reading to learn.

### Factors Influencing These Conceptions

This study unveiled a wider spectrum of teachers’ conceptions than did previous studies (e.g., Taboada and Buehl, 2012). The categories expanded from teachers focusing on helping their students to answer examination questions accurately only, to cultivating independent readers. This section applied the sense-making theory to analyze the historical–cultural background and social contexts in relation to the teachers’ conceptions as a portrait of the varying degrees of attainment of the objectives of the reading curriculum stipulated by the Hong Kong Education Bureau.

#### The Context: Historical–Cultural Background That Shapes Teachers’ Conventional Understandings

We examined teachers’ original understanding of learning from a historical perspective. Although simultaneously exposed to both Chinese and Western cultures (Chan and Elliott, 2002), the Hong Kong population was hardly influenced by the education culture of the British people (Berry, 2011) but only borrowed the assessment structure and had remained rooted in utilizing high-stakes examinations as their means of determining students’ prospects in life. Like most Confucian Heritage communities, Hong Kong inherited examination–orientation teaching in its education system. This influence of examination culture has permeated in the teachers’ conceptions of reading instruction.

Although teachers value curriculum initiatives like the Six Types, they are constrained by the largely examination-oriented learning culture in Hong Kong (Berry, 2011; CDC et al., 2015, p. 74), as seen in Category 1 teachers’ interview data. In order to be more assured of success in examinations, teachers focused on items that test discrete knowledge and spend a large amount of class time explaining vocabulary, rhetorical strategies, and main ideas of essays rather than facilitating students’ engagement in the reading processes.

The following two aspects explained the teachers’ conceptions as shown in Category 1. Firstly, the teachers put the accountability for students’ performance in examinations on themselves to a large extent (Berry, 2011; Tan, 2013). In order for their students to score well, they operate their reading classrooms with excessive test drilling. Research in Chinese language reading classes still sees a high occurrence of rote learning, a method both inherited from the traditional Chinese scholars and had propagated in an examination-oriented context (Tse, 2009). When given the opportunity to explore and expand learning, teachers choose to fall back to teach and drill the basic language skills as a safety net. Secondly, whether or not to execute certain reading activities relies on the possibility of operationalizing them as test items. Teachers are looking for standard answers they could give an objective grade to, even to the extent of favoring multiple-choice items in assessment. In the teachers’ mind, there seems to be a fixed course content that they need to cover so that their students will be able to provide “model answers” to the examination questions (Joseph Jeyaraj and Harland, 2019). Thirdly, as parallel to previous studies (e.g., Torff, 2005; Nazari and Bagheri, 2014), teachers tend not to perceive higher-order thinking activities as appropriate for every student. In the case of the current study, teachers in Category 1 considered the higher-order thinking skills as “too challenging” and “unachievable” by students who are of a younger age or with lower language proficiency. The deciding factor was based on the teachers’ assumptions of low performance, with the exception of *Teacher 502*, who opined that “Creating is attainable by my students, just that we need to acknowledge the extent that they can achieve.” With reference to Torff’s (2005) study, although it highlighted how in-service teachers, as compared with pre-service teachers, tend to believe that higher-order activities are more suitable for older or more proficient students, the current study further unveiled the reason for in-service teachers to avoid trying higher-order thinking skills with their less abled students. These teachers are under the impression that the information, knowledge, or experience that these students are sharing cannot meet the assessment criteria, and thus, they are less valued.

Despite the call to maintain a balance between learning, teaching, and assessment (CDC et al., 2015, p. 43), the teachers’ biggest concern is still at the high stake examination that brings the students to the next stage of their academic path and far from cultivating the students’ higher-order thinking skills. The negative washback of excessive testing in the educational context in Hong Kong is a societal focus of assessment that deviated the teachers’ interest to explore or expand their understanding of teaching and learning.

#### The Regulative Means: Messages About the Reading Curriculum

The implementation of competence-based education is not new to the teachers. The Chinese language curriculum was introduced together with level descriptors for the four language skills – reading, writing, listening, and speaking – which allowed teachers the flexibility to organize teaching materials (CDC, 2001). The level descriptors for reading competence helped to eliminate rote learning and prescribed texts (CDC, 2007, 2008), and they placed emphasis on higher-order thinking skills. Teachers are encouraged to organize the reading texts to actualize the goals prescribed in the level descriptors (HKEAA, 2005).

When the Six Types of Reading Comprehension Process Model was introduced, the Hong Kong Education Bureau (EDB) commissioned many practical workshops to facilitate the curriculum reform (Lau, 2007b, 2009). These regulative efforts of the reform explicitly influenced teachers’ conceptions (Coburn, 2001). To a large extent, the implementation of the Six Types interacted with the teachers’ conventional understanding that reading instruction is the teaching of the prescribed texts and shifted their focus from teaching content to cultivating skills. When the teachers were asked on their acclimation with Six Types, the following teachers responded as follows:

There is no need to [discuss with my colleagues], because in our examination setting, there is a table which already stated the different levels, and we all understood it. We will only discuss, say for Elaborating, what are the suitable issues that is currently of concern that we want to set a question for the level “Elaborating.” For example, if this year, we focused on “Appreciation,” so we may discuss among ourselves on setting a question that will be able to test students’ ability to elaborate on the topic Appreciation, and also discuss on how to add some content related to Appreciation in teaching. (Teacher 201)

Since I started teaching in this school, we focused on writing when we prepare our lessons. As for the Six Types, it can be easily adapted, since the textbooks had matched all their questions to the framework. (Teacher 301)

These teachers expressed that because this framework is already in use, there is no particular need to mention it or discuss among their colleagues; others have claimed that the Six Types was adopted in their instructional materials and adapted as assessment frameworks, which had become a natural frame of mind that they will apply in classroom teaching as a questioning technique.

Additionally, the current study discovered Category 2 and 3 teachers’ acceptance of creativity as an important skill to acquire and integrate with the teaching with other skills, and it further expanded to using the information read to complete learning tasks. As reported by the teachers, many found Creating as the most challenging part of reading comprehension, because students are required to internalize what they have read and then to generate new ideas. When Teacher 103 responded to creative reading tasks, she found her students either simply copied sentences from the reading materials or expressed their thoughts freely without making references to the source materials. From her point of view, both cases required teacher intervention, and the role of a teacher is to set clear boundaries for students to generate and express new ideas in a reasonable way.

*Students think they can answer freely in a creative task. But their responses are supposed to be text-based to some extent. Thus*,… *it is necessary to remind them that they should not do it that way*… *I think it requires more timely teacher feedback in creative tasks, but it makes no difference in terms of class preparation. (Teacher 103)*

With the introduction of Creating in the Six Types framework, we noted the cultivation of creative thinking requires teachers to provide additional guidance and to be more tolerant of the mistakes that students make in the learning process. We also acknowledged the challenge faced by the teachers in assessing it reliably.

#### The Process: Teachers’ Considerations in Responding to the Six Types

Adapting from the findings in Coburn’s (2001) study, we used the four domains: attainability by students, agreement with own philosophy, feasibility due to resources issue, and comprehensibility to analyze the teacher interview data in this study.

##### Attainability by students

Teachers in Category 1 reported that their students either are too young or are not capable to attain the Six Types. They claimed that their students need to build their foundation in Retrieving, Explaining, and Summarizing until they are matured enough for the higher-order thinking skills, that is, Elaborating, Evaluating, and Creating. *Teacher 801* explicitly divided the skills for the different levels, stating “lower primary will focus on lower-order skills, and for the sake of examination, higher primary students will focus on Evaluating and Creating.” Teacher 102 also claimed that for her lower primary students, explaining that “Evaluating is not impossible, but I will not frequently use it, as the students lack life experience and they cannot understand what they are doing, or the effect is just not ideal,” whereas Teacher 501 felt that “some of my students are capable of attaining other skills, but definitely not Creativity.” Parallel to previous studies (e.g., Torff, 2005; Nazari and Bagheri, 2014), teachers tend to perceive higher-order thinking activities as inappropriate for every student, as they deemed the performance as less valued because the information, knowledge, or experience that these students are sharing cannot meet their expectations.

##### Agreement with own philosophy

While sharing on their approaches in using the Six Types, teachers indicated that the implementation had influenced their beliefs in using a reading framework to cultivate skills and not relying on memorizing text. Teachers reported that they use the framework in their classroom questioning, in designing worksheets and class activities, and in supplementary materials. For example, *Teacher 603* used the Six Types as a guide when she is preparing her lessons, particularly while designing the guiding questions to be used during class discussion. While using instructional materials prepared by the school, *Teacher 901* applied the Six Types framework to examine for any missing important skill, or if her students need any intermediate scaffolding to reach a higher-level skill. Teachers in this study exhibited a relatively high acceptance level of the Six Types, with some being able to refine the framework to suit their teaching needs. While Lau’s (2007b) study found that there is a larger group of teachers who somewhat believe in a text-based and competence-based balance, it is not vivid in the current study. This observation may be interpreted as a hybrid of the teachers’ conventional conception and the implementation of the Six Types.

##### Feasibility due to resources issue

Teachers did not report on the lack of time issue as found in Coburn’s (2001) study, but they have shared about the opportunity for the collaboration of the teaching team and other stakeholders in developing instructional materials in schools. Teachers in three of the participating schools have mentioned how collaboration with the Education Bureau and teacher educators to prepare and evaluate lessons together had helped them gained a better understanding of the reading models. Triangulated with the schools’ documents on the professional development plans, we observed that the goals are stated as (1) ensuring that the higher-order skills are included, (2) collecting evidence on feedback for students, and (3) matching the skills to the difficulties faced by the students during comprehension.

##### Comprehensibility

No teachers claimed that they did not comprehend the Six Types, but in their descriptions, we observed that some teachers were unable to distinguish between Explaining and Evaluating, whereas some could not illustrate Retrieving and Summarizing well. Zhu et al. (2016) found that teachers have the highest concerns at the informational and collaboration stages in their study, and they implied that teachers will need to acquire new knowledge in these aspects. When describing a meeting that the school teachers were having on setting examination questions,

Our school wishes to set more questions on Evaluating and Creating. However, we found that the students scored better at Retrieve and Explain questions. In order for them to score better during examination, we set questions that only asked them to Explain. But as Evaluating requires them to use their own words and illustrate using own experience, our students are not able to perform well in an examination setting. (Teacher 902)

We observed a relatively high acceptance level by the teachers than did other studies (e.g., Coburn, 2001), with teachers still having concerns over the attainability by their students and agreement with their own philosophy, but less affected by resources and comprehensibility issues. This may be attributed to the following reasons. Firstly, the Six Types had been implemented widely by the Education Bureau and teacher educators for nearly two decades. Secondly, teachers with grade levels that are further away from major examinations welcome new initiatives more, as they face less stress.

## Implications, Limitations, and Conclusion

This study explored a hierarchical structure of categories of teachers’ understanding of reading comprehension processes, and its formulation through the lens of teachers’ sense-making theory. The three identified categories revealed that external demands may engender an oversimplified view of reading comprehension as an accumulation of linguistic and textual knowledge as in Category 1 and in turn constrain the development of higher-order thinking skills among primary school students. But if teachers connect higher-order thinking skills and reading comprehension as illustrated by Categories 2 and 3, they gradually improve their capacity in probing skills, giving feedback, and grading the students’ performance, even if the students are of a younger age or of lower language proficiency. The findings in this study also indicated that in order to promote conceptual changes among teachers, their beliefs will need to be brought to light with the confrontation of the inadequacy of those beliefs. Only then teachers will be given the opportunity to integrate new and old knowledge of teaching and learning.

Although much deliberation had taken place to ensure the authenticity of this study, like any other approaches, phenomenography has its inherent limitations in generalizing to a wider population (Åkerlind, 2012) and the extent to which researchers can bracket their own beliefs and previous results might interfere with the experience of the teachers. The study is also limited to understanding teachers’ reported conceptions, which more studies could further triangulate with other data from examining the relationship between teachers’ self-reported conceptions and their practice.

## Data Availability Statement

The datasets generated for this study are available on request to the corresponding author.

## Ethics Statement

The studies involving human participants were reviewed and approved by the Department of Chinese and Bilingual Studies The Hong Kong Polytechnic University. The patients/participants provided their written informed consent to participate in this study.

## Author Contributions

XZ led the research project in collaboration with CC with the assistance of GL and JW. All authors participated in the conception and execution of the project, and the manuscript was drafted collectively as well. CC and JW later worked collaboratively on the revision of the manuscript after the initial draft was completed while maintaining close communication with XZ and GL.

## Conflict of Interest

The authors declare that the research was conducted in the absence of any commercial or financial relationships that could be construed as a potential conflict of interest.
