# Supplementary material for: Primary School Teachers’ Conceptions of Reading Comprehension Processes and Its Formulation
Source: Front Psychol. 2020 May 7;11:615. doi: 10.3389/fpsyg.2020.00615 (PMC7221181; doi:10.3389/fpsyg.2020.00615)
Supplement: Supplementary file 1 [file Table_1.DOCX]

**Appendix A.** Interview Questions

| Questions | Purpose |
| --- | --- |
| 1. Please use the cards to organize the six skills from the Six Types of Reading Comprehension Process and elaborate on your choice. | This task allows participants to visually represent the six skills while helping them recall the Six Types and how these skills are used in their teaching practices. |
| 1. What is your practice and experience with the Six Types?    1. Do you use them in the classroom or when designing questions for assessment?    2. Would you refer to the Six Types when you are planning a lesson or designing a test paper?    3. How frequently do you apply the Six Types in your work?    4. Out of the Six Types, which one do you like using the most? Which one are you most comfortable with? Why?    5. Which one do you think your students prefer the most? Why? | To elicit teachers’ practice using the Six Types |
| 1. Were there challenges when you tried to use the Six Types in your teaching?    1. What are some problems faced by the students?    2. What are some problems faced by the teacher?    3. What problems did you overcome? How did you manage to do so?    4. If this certain aspect of the Six Types is so difficult to use, why are you still teaching and training students on this aspect of the Six Types? | To understand teachers’ concerns and difficulties when applying the Six Types in their work |
| 1. What is the purpose of using the Six Types?    1. What was of primary importance to you when you used the Six Types in assessment?    2. How well was the core purpose of the assessment achieved?    3. What are some additional benefits achieved besides the original purposes of the assessment?    4. Are all Six Types essential in Chinese language education? | To understand teachers’ motivation and purpose behind using the Six Types as a tool in their classrooms |
| 1. What kind of cognitive ability do you expect to see from your students?    1. Why are you using the Six Types? What is your purpose for using it? What do you want from it?    2. What do you expect to see in students’ capacities? What can be reflected in their work? | To elicit how teachers think the Six Types can be used to demonstrate the cognitive abilities and reading comprehension capacities of the students |
| 1. If you are monitoring a beginning teacher, what advice would you give them regarding the use of the Six Types?    1. What do they want the students to take away from their lessons?    2. What advice would you give them on the ideal intellectual outcomes with the Six Types?    3. What are some of the challenges they might encounter?    4. What is your advice to overcome these challenges? | To challenge teachers to think from an outsider perspective to elicit new insights |
| Closing: Is there anything else you would like to add to the discussion about the Six Types? *Interviewees are welcome to rearrange the cards from the beginning of the interview. | To elicit other comments the teachers would like to make on the Six Types |
